# Alterations in Hair Follicle Dynamics in Women

**DOI:** 10.1155/2013/957432

**Published:** 2013-12-24

**Authors:** Claudine Piérard-Franchimont, Gérald E. Piérard

**Affiliations:** ^1^Department of Dermatopathology, Unilab Lg, University Hospital of Liège, CHU Sart Tilman, 4000 Liège, Belgium; ^2^Department of Dermatology, Regional Hospital of Huy, 4500 Huy, Belgium; ^3^Laboratory of Skin Bioengineering and Imaging, Department of Clinical Sciences, B23, University of Liège, 4000 Liège, Belgium

## Abstract

Endocrine changes supervening after parturition and menopause participate in the control of sebum production and hair growth modulation. The ensuing conditions include some peculiar aspects of hair loss (effluvium), alopecia, and facial hirsutism. The hair cycling is of major clinical relevance because most hair growth disorders result from disturbances in this chronobiological feature. Of note, any correlation between a biologic abnormality and hair cycling disturbance does not prove a relationship of causality. The proportion of postmenopausal women is rising in the overall population. Therefore, the prevalence of these hair follicle disturbances is globally on the rise. Current therapies aim at correcting the underlying hormonal imbalances, and at improving the overall cosmetic appearance. However, in absence of pathogenic diagnosis and causality criteria, chances are low that a treatment given by the whims of fate will adequately control hair effluvium. The risk and frequency of therapeutic inertia are further increased. When the hair loss is not controlled and/or compensated by growth of new hairs, several clinical aspects of alopecia inexorably develop. Currently, there is little evidence supporting any specific treatment for these endocrine hair disorders in post-partum and postmenopausal women. Current hair treatment strategies are symptomatic and nonspecific so current researchers aim at developing new, targeted methods.

## 1. Introduction

The hair follicle represents a complex miniorgan consisting of different cell populations characterized by their distinct locations, functions, and molecular component expressions. It represents a uniquely dynamic system undergoing continuous growth cycles throughout life [1, 2]. This miniorgan normally regenerates itself 8 to 10 times during the human lifespan [3]. The cyclic hair follicle transformation process arises under the control of an oscillator system called “the hair cycle clock.” It occurs simultaneously with changes in the sebaceous gland, perifollicular dermis, and subcutis [3, 4].

A synchronized hair cycle prevails in most mammals preparing hair coat for seasonal environmental changes. In humans, hair growth is not a continuous process, but it follows a successive asynchronous rhythm leading to a periodic regeneration of hair follicles. The reason for this unsynchronized process in humans is unclear [5]. The hair cycle is controlled by various hormones and local growth factors probably produced both inside and nearby the hair bulb and follicular papilla [6]. In addition, minute environmental changes potentially affect the hair biology. Hair growth disorders are attributed, at large, to alterations in the normal dynamics of the hair cycle [1, 7]. The total density of hair shafts is then altered [1, 8].

Some of the clinical consequences of disturbed hair biology are gender dependent. A series of clinical presentations are quite specific at some periods of women's life. For each of these conditions, the pathomechanism appears quite distinct, allowing for expecting some future progress in their management. This review aims at raising awareness in the problems of hair disorders in postpregnant and menopausal women and discussing some assessment and treatment options pertinent to this group of women.

## 2. Basic Aspects of Hair Cycle Chronobiology

**Figure 1:**
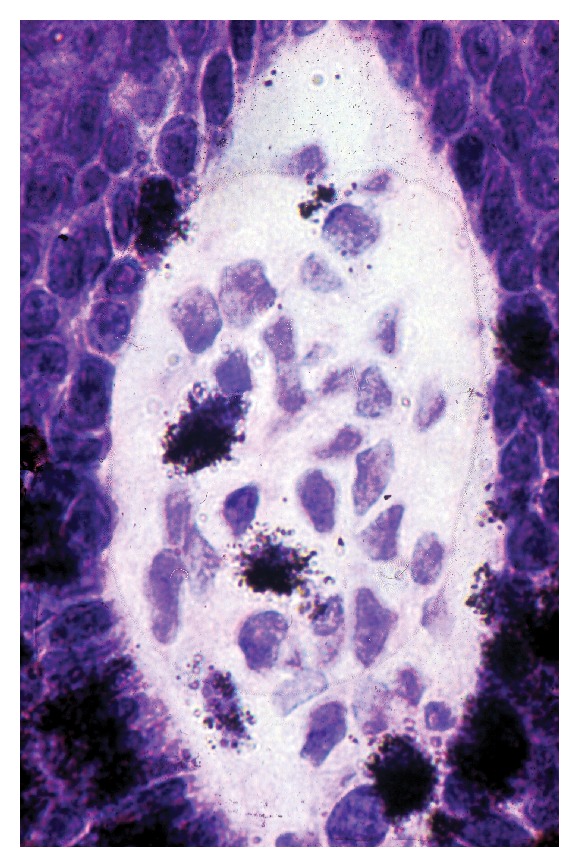
Growing hair in anagen phase with numerous cells in S phase of proliferation in the hair bulb and a few others in the dermal papilla. Tritiated thymidine autoradiography.

Clearly, there is a wide range of hair complaints in women, but it remains quite impossible to find relevant epidemiological data in the literature. Hair growth involves a unique process of cyclic regeneration from follicles undergoing phases of growth and rest [1, 3, 5, 7, 8]. The succession of the anagen, catagen, and telogen phases follows a recurrent period closely controlled by chronobiological synchronizers. The duration of the hair cycle is influenced by age, pathology, and a wide variety of physiological factors. The anagen phase duration of any individual hair ranges from about three to six years. This hair growth phase is characterized by intense cell renewal followed by terminal maturation. It involves epithelial growth of the hair bulb deep into the dermis with generation of a hair shaft (Figure 1). At completion of this growth phase, the hair bulb follows a rapid involution of the hair follicle during the catagen phase. The hair follicle retracts up to the mid part of the hair follicle and proceeds to the quiescent telogen phase. About 15 to 20% of scalp hairs are usually in telogen. After three months in average, a new hair bulb is expected at the base of the same follicle as the new successor anagen hair shaft. As it grows down, the old telogen hair is expelled. In such a process, each shed hair is ideally replaced by a new one. Although the actual mechanisms involved in hair shedding are not fully identified the process leading to the final hair shedding is possibly driven by the conjunction of activities of proteases and protease inhibitors [9–11]. At completion of the telogen phase, the club hair shaft remains loosely anchored to the follicular outer root sheath. The end of the telogen phase at the time of hair shedding corresponds to teloptosis/exogen phase [12, 13]. The hair falls spontaneously or is removed by gentle combing and washing. It is believed to be pushed out when the new anagen hair emerges. However, the process of hair shedding possibly occurs in humans regardless of the other hair cycle phases [1, 5].

The chronobiology control system driving the cyclic process in the hair follicle remains putative. Controls of the onset and duration of any hair cycle phase are complex. They possibly involve a series of up- and downregulated synchronizers. The whole process sustains hair growth moving from one growth stage in the hair cycle to the next [12].

During hair cycling, a series of cell types and structures are involved. They include endothelial cells, lymphocytes, basement membranes, proteoglycans, and the abutted constitutive cells of the epidermis, dermis, and hypodermis [14]. They possibly influence the cycle. Whether some of these changes represent causative or concomitant features is not established. The most obvious driving force in the hair cycle appears issued from both the follicular papilla and the perifollicular matrix. Indeed, prominent structural changes occur in these structures during the hair cycle. The follicular papilla is large in established anagen. It becomes smaller and more compact in telogen. The size of the follicular papilla dictates the size and shape of the follicle [14–16] which are particularly dictated by the body location. The variable papilla dimensions during the hair cycle are reflected in the extracellular matrix, remodelling with accumulation of proteoglycans including versican [17]. The vascular structures become more prominent during the growth phase. Factors from the papillary mesenchymal cells probably act on the follicular epithelium. At initiation of the anagen phase, factors from the papilla influence stem cells of the hair follicle that are receptive to inductive signals [18].

## 3. Coordination between Successive Hair Cycles

In subjects with reduced hair density, most follicles contain only a single hair, and some others appear empty [19]. By contrast, in hair fullness conditions, follicular openings commonly contain a couple of hairs. This aspect is possibly due to several hair follicles sharing in common a single infundibulum. Delayed teloptosis further influences such pattern [13]. Teloptosis can occur concurrently as the hair follicle initiates the next early anagen stage or has already been in anagen for some time.

By contrast, in some conditions the telogen hair is lost before the next anagen hair becomes visible. During the latency period, the hair follicle appears empty at the clinical inspection [20–22]. The lag time before the extrusion of the next generation of hair corresponds to the hair eclipse phenomenon [22]. This physiological feature is more or less prolonged, reaching 4 to 7 months in average [20, 23]. It obviously influences the hair density of the scalp. The hair eclipse phenomenon likely depends on a series of distinct synchronizers. It results from some dysregulations in the hair cycling involving early teloptosis, delayed anagen initiation, or stunted hair growth at any step during the anagen stage. As such, the hair eclipse phenomenon presents as an erratic process occurring in a series of physiopathological conditions affecting hair follicles singly or in focal to generalized patterns. It is more frequent following synchronized teloptosis occurring in telogen effluvium such as a postpartum alopecia. Local synchronizers including growth factors and other mediators are eventually lacking or involved in the hair eclipse phenomenon. Their identification and characterization possibly drive new corrective and preventive applications.

The underlying causative mechanisms of generalized hair eclipse phenomenon are probably diverse. They remain unsettled. Two typical examples are given by the arrest of hair growth during the midanagen phase in alopecia areata and in the transient baldness occurring in some newborns or after chemotherapy. The chronic telogen effluvium [21] is similarly followed by the hair eclipse phenomenon. The diversity of causes and mechanisms involved in the hair eclipse phenomenon is remarkable [5, 20].

## 4. Postpartum Hair Loss

Most women suffer from telogen effluvium during postpartum [1, 24, 25]. Indeed, during pregnancy, the teloptosis/exogen phase is delayed and the number of shedding hairs is reduced, inducing increased hair fullness. After delivery, the hair cycles become synchronized on the scalp of these women ending in a synchronous telogen-teloptosis process [26]. At that time, hair loss appears abundant but only represents the elimination of the additional hairs that had been maintained in anagen phase and had not been lost during gestation. In the second and third quarters of pregnancy only about 10% of hairs are in telogen.

Postpartum hair loss is commonplace and considered as a minor nuisance by women who have previously passed through it. However, it possibly represents a panic condition for these who experience it for the first time. The same process operates following severe febrile conditions. During the first weeks of postpartum, hairs enter telogen in a synchronous wave to reach about 30% in average after nine weeks. This explains the clinical observation that postpartum hair loss is experienced two to four months after childbirth. It usually continues for 6 to 24 weeks but rarely persists up to 15 months.

Postpartum hair loss from the scalp is diffuse but is accentuated along the anterior hair line. Virtually the whole hair is replaced after several weeks unless some other process unmasks female pattern alopecia.

## 5. Hair Follicles during Climacteric

The average age at natural menopause is about 49–51 years. The hair follicles are often affected at that period of life. Indeed, hair represents a specific receptor structure expressing declines related to fluctuations in circulating levels of sex steroids. The two major changes in hair distribution seen at menopause are female pattern alopecia (FPA) and facial hirsutism. Both conditions are often concomitant and seem to be more pronounced with increasing postmenopausal years. Tibolone, as an alternative to hormone replacement therapy (HRT), has been reported to increase the severity of diffuse alopecia and possibly induce facial hypertrichosis [27]. Frontal fibrosing alopecia (FFA) is a distinct condition probably accentuated by menopause, although it is not controlled by HRT.

## 6. Climacteric Hair Loss

Most women in developed societies expect to spend a third or more of their lives after menopause. Aging associated with climacteric hormonal changes commonly affects some hair characteristics and is responsible for decreased hair coverage in middle-aged women [28]. The most common form of progressive alopecia in elderly women is FPA, which often worsens during the perimenopause, particularly if the condition was previously present [29, 30]. The decreased anagen phase duration and regression of scalp hair to finer vellus hair are particularly caused by androgens. They commonly lead to climacteric alopecia [23]. Of note, some women presenting with alopecia do not show any increase in androgen circulating levels, suggesting that their androgen skin receptors are abundant or that other androgen-independent mechanisms are involved [29]. FPA particularly affects hair follicles from the parietal and frontosagittal areas causing bitemporal hair thinning but leaving an intact frontal hairline [29, 31]. In this condition, both the scalp stratum corneum and the hair shafts show a decreased capacitance level indicating an impaired moisturization [32, 33].

Postmenopausal FFA is a distinct cicatricial alopecia [34–36]. It corresponds to a progressive condition responsible for the destruction of the upper portion of the hair follicle by a lymphoid cell infiltrate. This process induces a distinctive pattern of hair rarefaction corresponding to a symmetrical regression of the frontal and temporal hairline, combined with partial to complete loss of the eyebrows [34–36]. The hair loss onset is particularly difficult to identify as patients present relatively late during the disease progression. Hence, FFA is often stable and this impacts the treatment efficacy [25, 36]. In addition, it remains unsettled how climacteric changes cause this selective targeting of the frontotemporal scalp. However, some benefit has been reported in a few patients following androgen-dependent therapy [24, 26, 35].

## 7. Pregnancy and Climacteric Facial Hirsutism

Increased hair growth during pregnancy is a rare condition associated with signs of virilization such as deep voice, acne, and clitoral enlargement. In some of these patients an arrhenoblastoma was disclosed, and in other women bilateral ovarian enlargement results from lutein cell hyperplasia. Virilization possibly disappears during postpartum in association with a spontaneous decrease in the ovarian size.

Body hair in women usually tends to rise until menopause. Later on, it begins to decrease. By contrast, facial hair tends to increase even in the elderly. The prevalence of hirsutism in postmenopausal women has not been fully documented [37]. About 50% of women report excessive facial hair growth after menopause [38]. This feature suggests that both the clinical and social importance of this problem is underestimated in many instances.

In general, hirsutism is due to increased androgen production or to greater sensitivity of the hair follicles to these hormones [39]. Androgens are responsible for increasing the hair follicle size, hair shaft diameter, duration of the anagen phase of terminal hairs, and sebum production. All these aspects are typically present in hirsutism [40]. The vast majority of hirsute women suffer from a benign causal disorder. The two main causes are polycystic ovary syndrome (PCOS) and idiopathic hirsutism with circulating androgen levels within normal range [41–45]. Although PCOS and the associated hormone imbalances were observed after menopause, the level of hyperandrogenism appeared modest.

More serious but rare causes of hirsutism include the congenital adrenal hyperplasia and Cushing's syndrome, as well as some benign and malignant androgen-secreting ovarian and adrenal tumours [38, 46, 47]. Hyperandrogenism caused by adrenal ganglioneuroma [48] and ovarian-related hyperandrogenism associated with hirsutism including those related to ovarian hyperthecosis [49–51] and ovarian neoplasms [52] have been reported. Severe virilisation in postmenopausal women with nonmalignant origin is rare. Occasionally, postmenopausal hirsutism follows androgen therapy including testosterone [53] and androgen-estrogen hormone therapy [54].

## 8. Conclusion

By stressing the uniqueness of some hair disorders in women, those diseases and their treatment should come more sharply into focus. The ultimate goal is to better prevent, diagnose, and treat the hair disorders in women. The causes of hirsutism and hair loss as well as the management of these conditions are occasionally different in postmenopausal women compared with women of younger reproductive age [55]. Some postmenopausal women probably deserve special care and attention when entering the new phase in their lives supported by complex physiological changes. Chronobiology governing the hair cycle is a fascinating and complex process.
